# *ClusterFinder*: a fast tool to find cluster structures from pair distribution function data

**DOI:** 10.1107/S2053273324001116

**Published:** 2024-02-29

**Authors:** Andy S. Anker, Ulrik Friis-Jensen, Frederik L. Johansen, Simon J. L Billinge, Kirsten M. Ø. Jensen

**Affiliations:** a Department of Chemistry and Nano-Science Center, University of Copenhagen, 2100 Copenhagen Ø, Denmark; b Department of Computer Science, University of Copenhagen, 2100 Copenhagen Ø, Denmark; c Department of Applied Physics and Applied Mathematics Science, Columbia University, New York, NY 10027, USA; Helmholtz Centre for Infection Research, Germany

**Keywords:** pair distribution function analysis, nanoclusters, nanomaterials, screening

## Abstract

A novel automated high-throughput screening approach, *ClusterFinder*, is reported for finding candidate structures for atomic pair distribution function (PDF) structural refinements. Finding starting models for PDF refinements is notoriously difficult when the PDF originates from nanoclusters or small nanoparticles. The reported *ClusterFinder* algorithm can screen 10^4^ to 10^5^ candidate structures from structural databases such as the Inorganic Crystal Structure Database (ICSD) in minutes, using the crystal structures as templates in which it looks for atomic clusters that result in a PDF similar to the target measured PDF. The algorithm returns a rank-ordered list of clusters for further assessment by the user. The algorithm has performed well for simulated and measured PDFs of metal–oxido clusters such as Keggin clusters. This is therefore a powerful approach to finding structural cluster candidates in a modelling campaign for PDFs of nanoparticles and nanoclusters.

## Abstract 2

An automated high-throughput screening approach is presented for identifying starting structure models for pair distribution function analysis of nanoclusters.

## 1. Introduction

Throughout the last century, crystallographic methods have played a crucial role in advancing materials science, yet they often struggle when examining nanomaterials with limited long-range order (Billinge & Levin, 2007 ▸). Total scattering with pair distribution function (PDF) analysis has shown promise for characterizing such nanomaterials (Billinge & Levin, 2007 ▸; Christiansen *et al.*, 2020 ▸). The PDF, derived from the Fourier transform of normalized and corrected X-ray, neutron or electron scattering intensities, offers a real-space representation of interatomic distances in the sample. As the data used in the Fourier transform include both Bragg and diffuse scattering, PDF analysis can be used to characterize the structure of materials with or without long-range atomic order (Egami & Billinge, 2012 ▸; Christiansen *et al.*, 2020 ▸).

The challenge of *ab initio* structure solution from PDFs has long been pursued (Juhás *et al.*, 2006 ▸, 2008 ▸, 2010 ▸; Cliffe *et al.*, 2010 ▸; Cliffe & Goodwin, 2013 ▸; Anker *et al.*, 2020 ▸; Kjær *et al.*, 2023 ▸; Kløve *et al.*, 2023 ▸). However, success remains limited to rather simple chemical systems like simple inorganic crystals, the C_60_ buckyball and mono-metallic nanoparticles. In the absence of broadly applicable *ab initio* structure solution methods, suitable starting models are necessary to refine the PDFs. For crystalline or nanocrystalline materials, such starting models can, in many cases, easily be identified from crystallographic databases. However, this task becomes exceptionally difficult for small clusters and nanomaterials with significant disorder. Recent methods such as *Cluster­Mining* (Banerjee *et al.*, 2020 ▸), *StructureMining* (Yang *et al.*, 2020 ▸) and *POMFinder* (Anker *et al.*, 2024 ▸) have taken the approach of screening large numbers of structures that are pulled from databases or algorithmically generated. Nonetheless, they are all restrained to the presence of a suitable database of structures or an algorithmic structure generator.

We recently presented a hybrid approach, *ML-MotEx* (Anker *et al.*, 2022 ▸), where the user initially selects candidate crystal structures from a crystallographic database. Explainable machine learning is then used to find sub-clusters from the candidate structure that are consistent with the data, which can then be used for further structure refinement and analysis. The approach works well but is slow, taking several minutes for each starting structure. This limits its application to cases where the candidate parent crystal structures are few and obvious to the user. Here, we propose a novel algorithm, *ClusterFinder*, that follows the same approach of sampling sub-clusters from larger structural candidates, but it uses a non-machine learning direct-scoring approach for identifying high-performing sub-clusters. This speeds up the selection procedure from minutes to seconds, allowing for an automated search for sub-clusters over large numbers of candidate parent structures that can be selected in an automated way from structural databases.

## 2. Method

**Figure 1:**
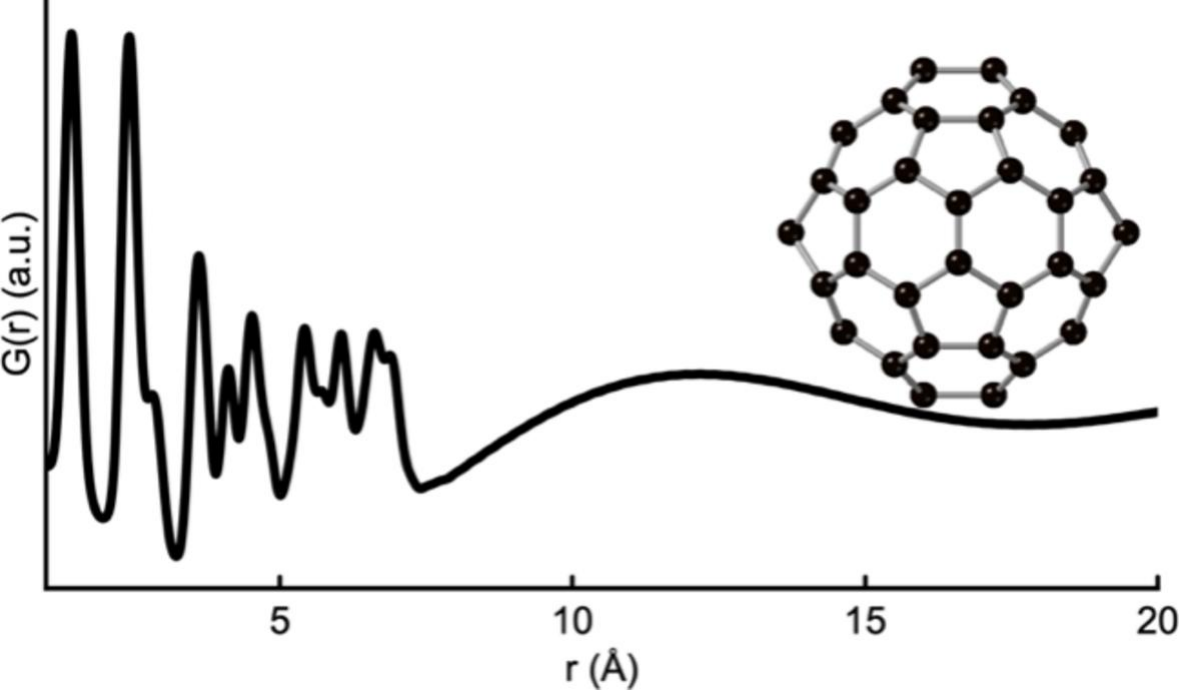
A simulated PDF for a C_60_ buckyball from a single unit cell of a C_60_ crystal structure (Chen & Yamanaka, 2002 ▸). The simulation parameters mimic typical PDF dataset values and can be seen in Section A in the supporting information.

The basic strategy for finding clusters from crystalline fragments was described by Anker *et al.* (2022 ▸). We summarize it here. The starting point is an atomic PDF experiment of a sample that contains small clusters, for example a soluble reagent or nanoparticles suspended in a solvent. The atomic arrangement in highly disordered materials can also sometimes be described using cluster structures (Du *et al.*, 2012 ▸; Castillo-Blas *et al.*, 2020 ▸; Christiansen *et al.*, 2020 ▸). The resulting measured PDF has a small number of peaks confined to the low-*r* region, indicating the presence of unknown atomic clusters of small size (see Fig. 1 ▸).

In principle, the data can be fitted using the Debye scattering equation in programs such as *DISCUS* (Proffen & Neder, 1997 ▸, 1999 ▸) or *DiffPy-CMI* (Juhás *et al.*, 2015 ▸) to understand the structure of the clusters, but this process requires a good initial candidate structure to be given. The main challenge is to find a set of good starting models for the fit. *ClusterFinder* addresses this need. It reuses the approach taken by *ML-MotEx* (Anker *et al.*, 2022 ▸) where a set of chemically reasonable crystal structures is first identified. From the crystal structures, which are represented using crystallographic information files (CIFs), candidate templates are then cut out. The candidate templates are represented in *xyz* format (a list of atomic identities and their respective Cartesian coordinates *x*, *y* and *z*). Assuming for now that the cluster present in the experimental data, the target cluster, is contained within the candidate template, the principal goal is to find the subset of occupied atom sites within that template that corresponds to the target cluster. A search over all possible permutations of present versus absent atoms is impossible because of the combinatorics, with 2^*N*^ − 1 possibilities for a template of *N* sites. *ML-MotEx* uses an explainable machine learning approach to optimize this problem by learning the probabilities that each atom might be present in the target cluster after iterating over a small subset of all the possible permutations. This places the atom sites in a rank-ordered list and makes it easy for the user to select a cut-off for which sites are occupied to generate the target cluster configuration. Of course, the target cluster may not be present in the template and in general there is a further outer loop that needs to be iterated over all possible candidate crystal structures and templates. The *ML-MotEx* algorithm is too slow to do this over many template candidates and the success of the approach relies on a strong chemical intuition suggesting a small number of candidate structures.

At the heart of the algorithm is the calculation to generate an ordered list of sites based on the probability that they are present in the target cluster. The *LIGA* algorithm (Juhás *et al.*, 2006 ▸, 2008 ▸) also scores atoms in a cluster as part of its backtracking cluster reduction step, where poor performing clusters are reduced in size by preferentially removing atoms that are contributing more error to the agreement with the data. The ranking was done using the commonly used PDF weighted profile agreement factor,

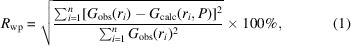

where *G*_obs_ and *G*_calc_ are the observed and calculated PDF intensities, respectively, for the set *P* of model refinement parameters. The sum is over the *n* points in the PDF.

Taking inspiration from the *LIGA* algorithm, we attempt an approach of computing the contribution to the fitting error for each atom site in the cluster. We call this the atom-removal error, and denote it for the *i*th atom by 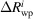 . It is computed by evaluating *R*_wp_ for the full set of atoms, then recomputing *R*_wp_ for the cluster with the *i*th atom removed and taking the difference. This allows us to identify which atoms contribute the most error to the fit, allowing us to target them for removal. For each computation of *R*_wp_, a scale factor and an isotropic expansion/contraction factor are allowed to be refined to give the best agreement. Atomic displacement parameters (ADPs) were fixed to 0.3 Å^2^ for the metallic atoms and 0.4 Å^2^ for the oxygen atoms. This procedure is extremely rapid and results in a list of atomic sites ranked by 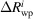 .

The candidate structure must be large enough to encapsulate the target cluster, but the computational cost scales linearly with the number of atoms of the template structure and so the cluster size chosen can thus be a compromise between time and the cluster structures screened.

**Figure 2:**
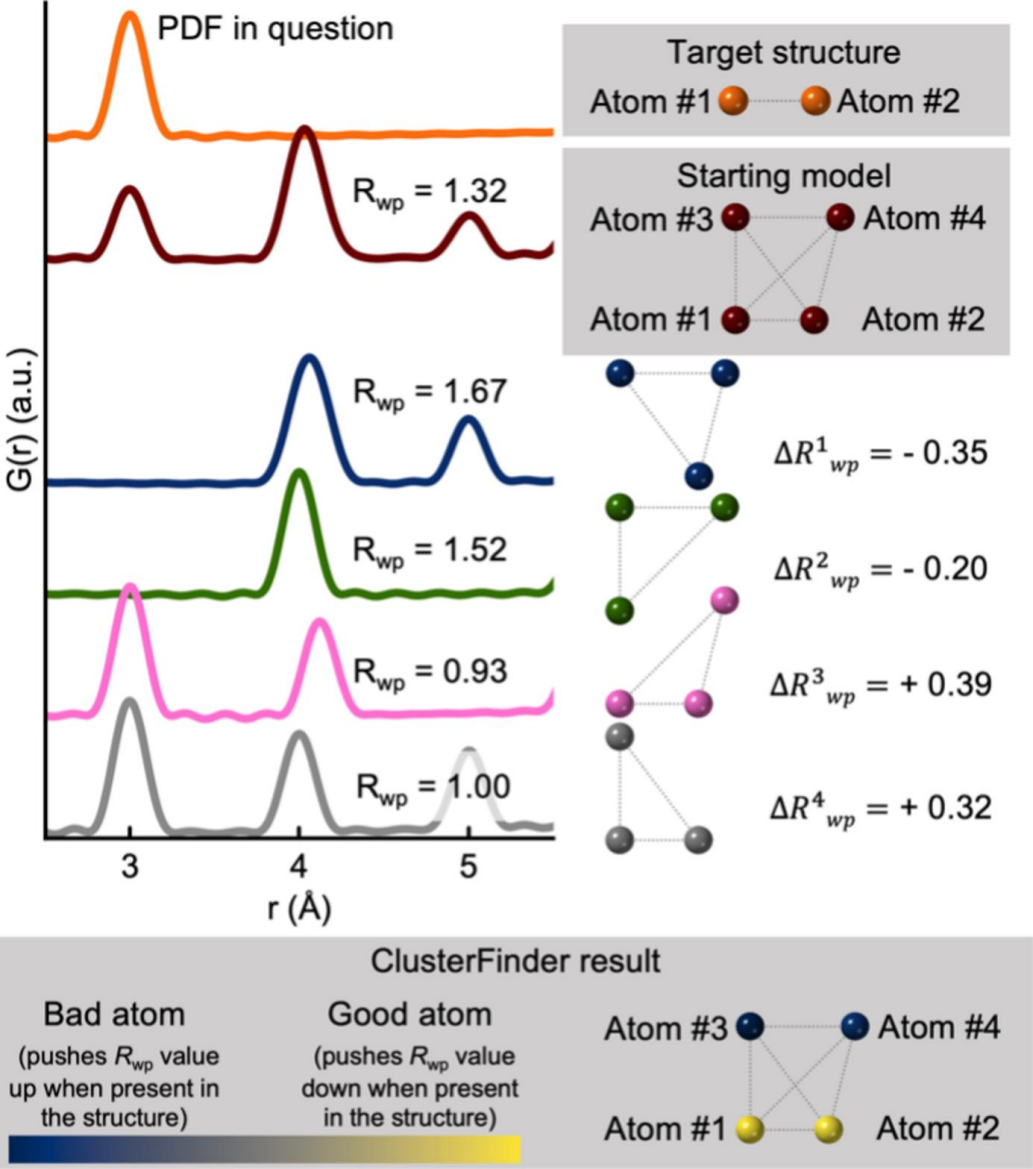
An illustration of the *ClusterFinder* process. A starting model is provided as input and the *R*_wp_ value is calculated by structure refinement. Atoms are iteratively removed from the starting model and the revised model is fitted to the experimental PDF. The atom-removal error 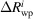 is calculated by taking the difference between the *R*_wp_ values of the full starting model and when the atoms are removed. Atoms are colour coded based on the atom-removal error – yellow indicates a negative 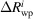 value (improved fit) while blue signifies a positive 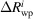 value (worsened fit).

To visualize the results, we plot the templates with each atom site colour coded based on its 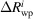 . Atom sites with negative (good) 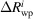 are coloured yellow and those with positive (bad) 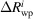 are coloured blue. The colour coding is further explained in Section B in the supporting information. The approach is illustrated schematically for a trivial example of a small cluster consisting of two atoms in Fig. 2 ▸. Note that *ClusterFinder* only ranks the atoms in the template, and a human input is still needed to determine which atoms to remove in the subsequent task of finding the best cluster candidates. In Fig. 2 ▸, it is trivial to remove atoms 3 and 4 from the *ClusterFinder* output, but this task might not always be trivial and may rely on the chemical intuition of the user. However, it is still extremely valuable because, due to its speed, it can be used to screen large numbers of structures to find the best cluster candidates.

To test the *ClusterFinder* approach, we here use it on simulated and experimental PDF data. *ClusterFinder* provides comparable results to *ML-MotEx* in quality but orders of magnitude more quickly. The acceleration is sufficient to allow screening of large databases of starting models in minutes. To demonstrate the power of this, we provide five examples where we screen the Inorganic Crystal Structure Database (ICSD, https://icsd.fiz-karlsruhe.de/index.xhtml; Zagorac *et al.*, 2019 ▸), containing 188 631 structure entries, for a suitable starting model. This is done in a time frame ranging from 3 to 42 min. We expect this to make *ClusterFinder* highly valuable since, if the target cluster exists anywhere in any known crystal structure, it will automatically be found without any user input at this stage.

## 3. Results and discussion

### 3.1. Applying *ClusterFinder* to extract cluster motifs from simulated PDFs

**Figure 3:**
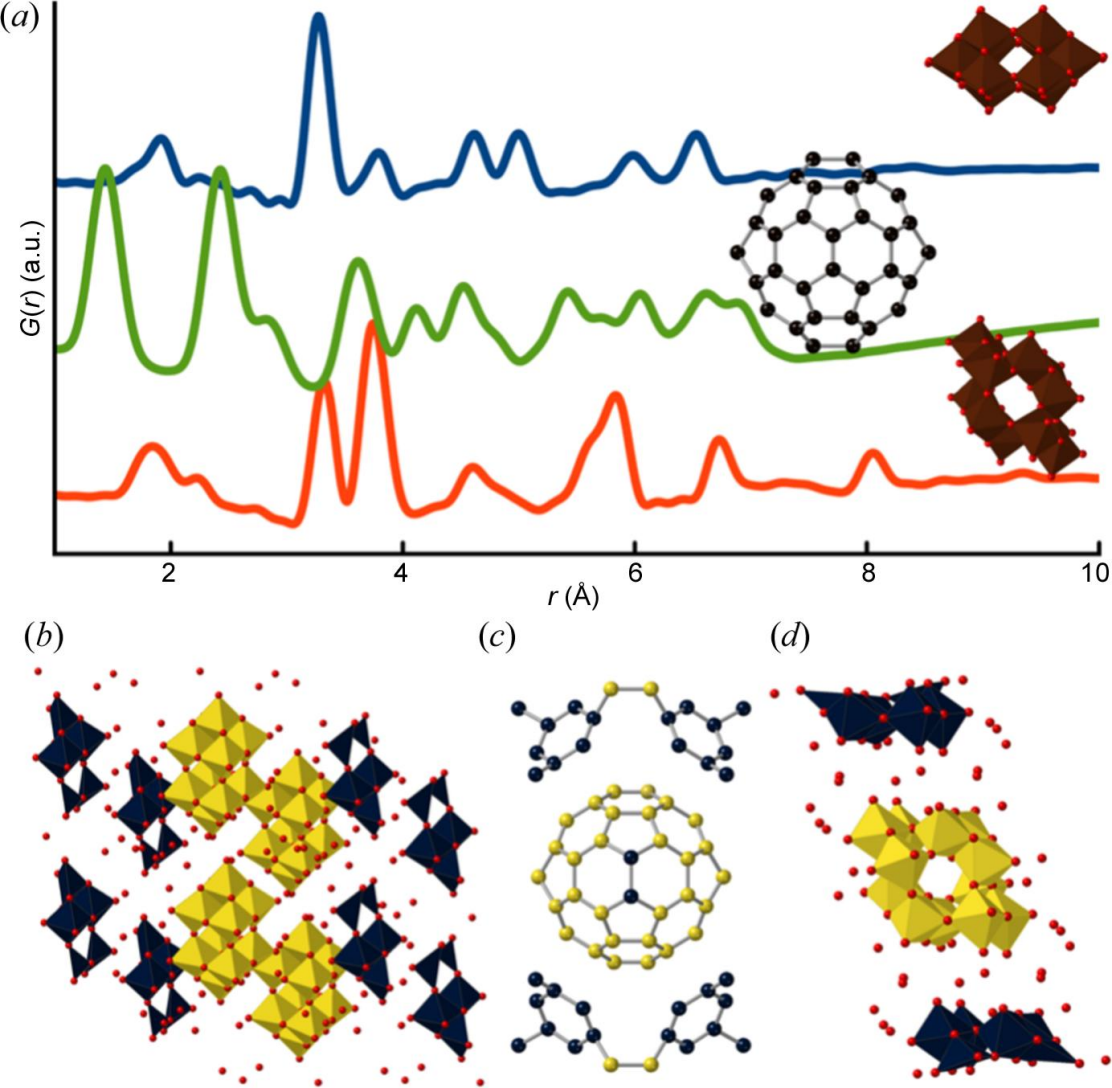
Analysis of simulated PDFs of well known cluster structures. (*a*) Simulated PDFs of a decatungstate polyoxometallate cluster from the Na_5_(H_7_W_12_O_42_)(H_2_O)_20_ crystal structure (blue) (Redrup & Weller, 2009 ▸), a C_60_ buckyball from a single unit cell of a C_60_ crystal structure (green) (Chen & Yamanaka, 2002 ▸) and a paratungstate polyoxometallate cluster obtained from the (Ba(H_2_O)_2_{H[N(CH_3_)_2_]CO}_3_)_2_(W_10_O_32_){H[N(CH_3_)_2_]CO}_2_ crystalline model (red) (Poimanova *et al.*, 2015 ▸). Simulation parameters were chosen to mimic typical measured PDF datasets and are listed in Section A in the supporting information. (*b*)–(*d*) Results of using *ClusterFinder* on the three simulated PDFs where the atoms with the (*b*) 40, (*c*) 60 and (*d*) 12 atoms with the lowest 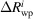 values have been coloured yellow, while the rest are coloured blue. Section C in the supporting information shows a similar representation but where the atom-removal values are directly shown using a continuous colour bar. Oxygen atoms are coloured red and polyhedra are coloured according to their metal atom centre.

We first demonstrate *ClusterFinder*’s ability to extract cluster motifs from simulated PDFs. Fig. 3 ▸ shows three simulated PDFs, each corresponding to a distinct structure: a decatungstate polyoxometallate cluster from an Na_5_­(H_7_W_12_O_42_)(H_2_O)_20_ crystal structure (Redrup & Weller, 2009 ▸), coloured in blue; a C_60_ buckyball from the C_60_ crystal structure (Chen & Yamanaka, 2002 ▸), coloured in green; and a paratungstate polyoxometallate cluster originated from a (Ba(H_2_O)_2_{H[N(CH_3_)_2_]CO}_3_)_2_(W_10_O_32_){H[N(CH_3_)_2_]CO}_2_ crystalline model (Poimanova *et al.*, 2015 ▸), coloured in red. The values of the simulation parameters used are listed in Section A in the supporting information. Figs. 3 ▸(*b*)–3 ▸(*d*) show the structural templates used by *ClusterFinder*.

In these tests, the structural templates were constructed using the crystal structures containing each of the cluster structures, and including the minimum number of unit cells needed to include the full cluster (Section C in the supporting information). *ClusterFinder* outputs a list of atomic sites ranked by the 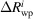 value, and we visualize atom sites with negative 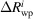 as yellow and those with positive 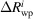 as blue. The ranking is here done on the metal atoms, while oxygen atoms are removed if they are beyond a distance threshold of 2.6 Å from any other atom. The resulting visualizations are shown in Figs. 3 ▸(*b*)–3 ▸(*d*), where the atoms with the lowest 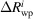 values have been coloured yellow, while the rest are coloured blue. Section C in the supporting information shows a similar representation but where the atom-removal values are directly shown using a continuous colour bar. Oxygen atoms are coloured red and polyhedra are coloured according to their metal atom centre.

*ClusterFinder* correctly extracted all three cluster structures from their starting model in under a minute using a standard laptop (Intel Core i7-8665U CPU at 1.9/2.11 GHz), demonstrating a significant speed advantage over the *ML-MotEx* algorithm (Anker *et al.*, 2022 ▸), which takes approximately an hour on the same computer. Although *ClusterFinder* accurately extracts the decatungstate polyoxometallate cluster (blue) and the paratungstate polyoxometallate cluster (red), it does not completely recover the C_60_ buckyball (green), incorrectly labelling two atoms. The *ML-MotEx* algorithm also exhibited similar limitations in extracting this structure. Note that while *ClusterFinder* is faster than *ML-MotEx*, the latter algorithm is more versatile and has, for example, also been used to determine stacking fault size domain distributions from experimental powder diffraction and PDF data from γ-MnO_2_ nanoparticles (Magnard *et al.*, 2022 ▸).

### 3.2. Applying *ClusterFinder* to extract cluster motifs from experimental PDFs

While *ClusterFinder*’s potential to extract cluster motifs from various crystalline supercell structures has been demonstrated with simulated PDFs, it must also work on experimental data. Here we benchmark the performance of *ClusterFinder* against that of the previously published *ML-MotEx* algorithm by comparing its performance on the same set of experimental PDFs and clusters.

An experimental PDF was obtained from a solution of 0.05 *M* ammonium metatungstate hydrate, (NH_4_)_6_­(H_2_W_12_O_40_)·H_2_O in water, which dissolves to form monodisperse α-Keggin clusters (Juelsholt *et al.*, 2019 ▸). Experimental details can be found in the *ML-MotEx* paper (Anker *et al.*, 2022 ▸). We employed four different crystallographic models to extract templates for *ClusterFinder*/*ML-MotEx* as listed in Table 1 ▸.

**Figure 4:**
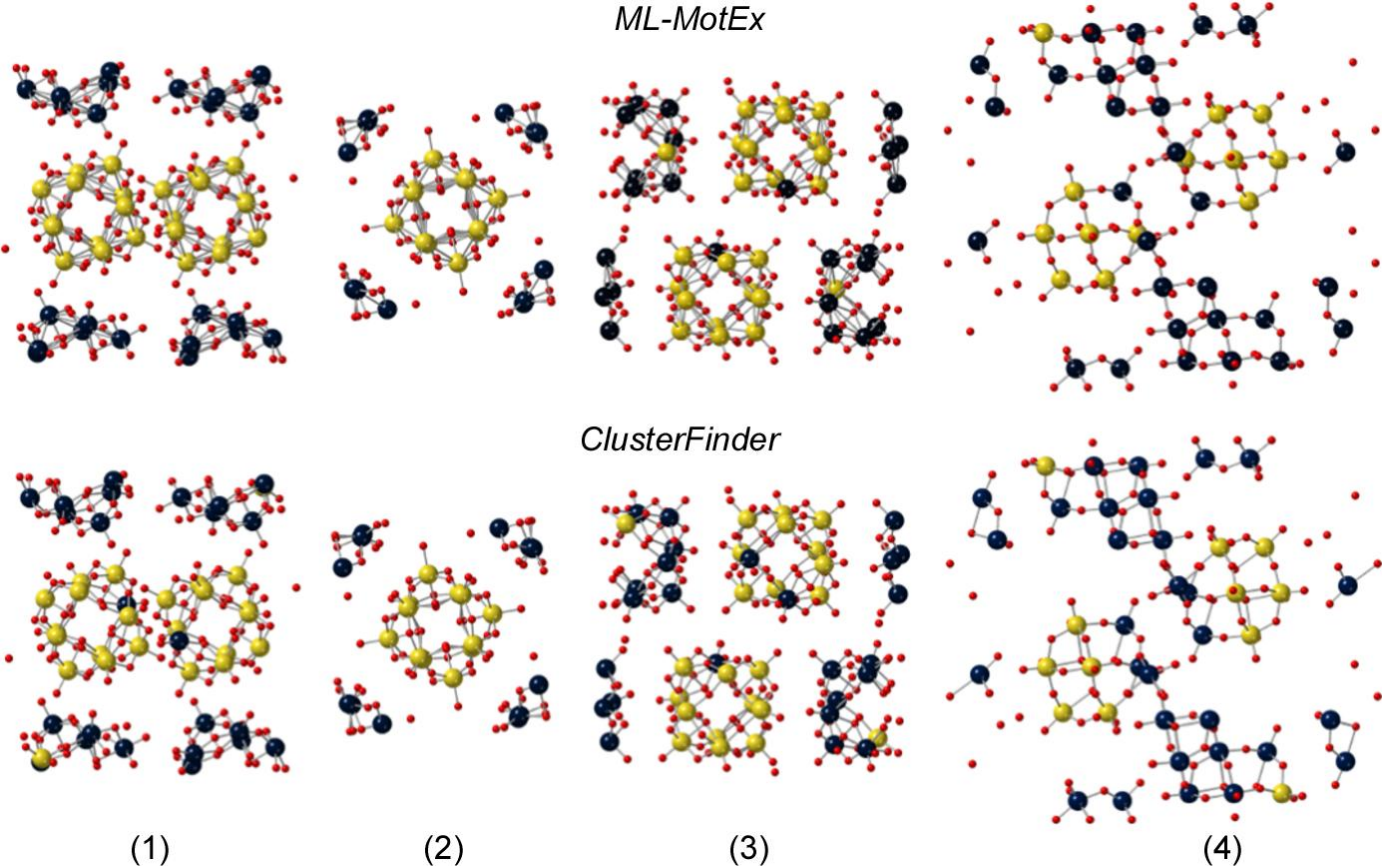
Comparisons of the *ML-MotEx* and *ClusterFinder* analyses of an experimental PDF obtained from Keggin clusters in solution. Results are given from the *ML-MotEx* and *ClusterFinder* methods on a PDF obtained from a solution of ammonium metatungstate hydrate using four different starting models, (1) (Hpy)_4_H_2_(H_2_W_12_O_40_) (py = pyridine) (Niu *et al.*, 2004 ▸), (2) [(CH_3_)_4_N]_4_SiW_12_O_40_ (Joachim *et al.*, 1981 ▸), (3) ([(CH_3_)_2_NH_2_]_6_{Cu[HCON(CH_3_)_2_]_4_}(GeW_12_O_40_)_2_)[HCON(CH_3_)_2_]_2_ (Niu *et al.*, 2003 ▸) and (4) [(CH_3_)_2_NH_2_]_3_(PW_12_O_40_) (Busbongthong & Ozeki, 2009 ▸). The 24 [structures (1), (3) and (4)] and 12 [structure (2)] atoms with the lowest atom-removal values have been coloured yellow, while the rest are coloured blue. Oxygen atoms are coloured red.

Again, only a scale factor and an isotropic expansion/contraction factor were refined during the *ClusterFinder* process. As seen in Fig. 4 ▸, both *ClusterFinder* and *ML-MotEx* successfully extracted the α-Keggin clusters with few mislabelled atoms for all four starting models. *ClusterFinder* has slightly more mislabelled atoms than *ML-MotEx*, but it is orders of magnitude faster, making it an ideal choice for screening larger databases.

### 3.3. Screening the ICSD for a suitable starting model with *ClusterFinder*

**Figure 5:**
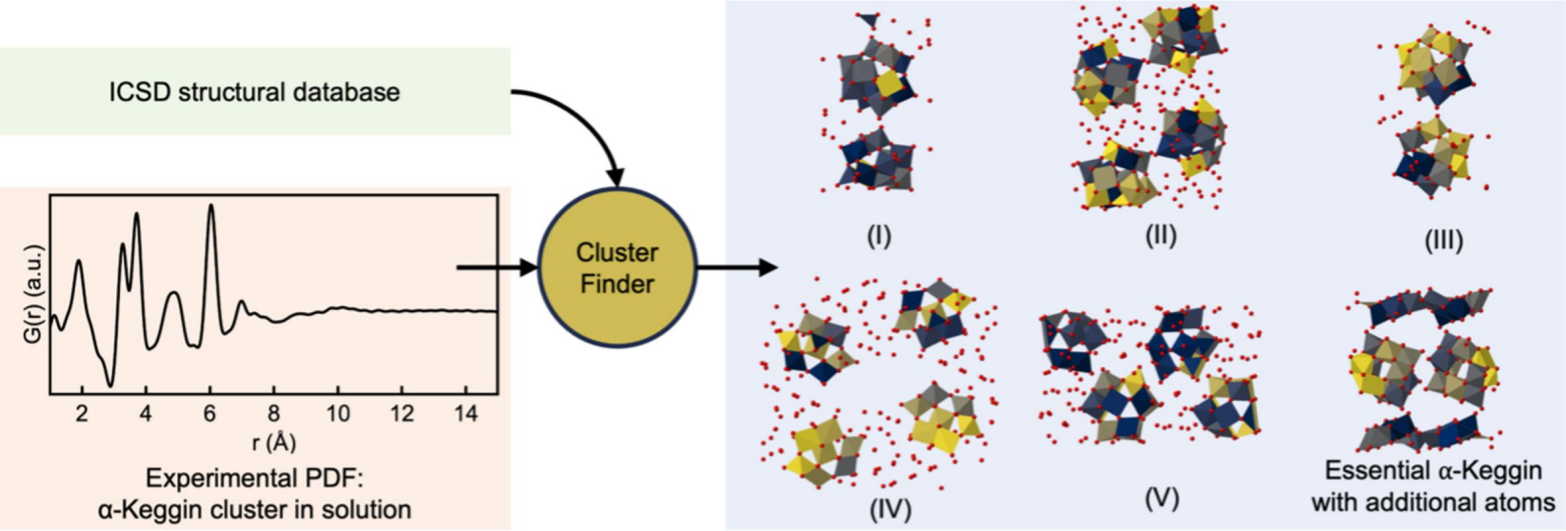
An illustration of how *ClusterFinder* is used to screen the ICSD for the correct starting model for an experimental PDF obtained from α-Keggin clusters in solution. For each structure in the ICSD, the *ClusterFinder* procedure is performed, and the atoms are colour coded based on their impact on fit quality using a continuous colour bar. Afterwards, the ICSD structures are sorted according to their average 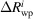 values. The five candidate ICSD structures with the lowest average *R*_wp_ value are highlighted. The top five candidates are all starting templates exclusively comprising essential cluster structures – clusters in which no atoms need removal and that thereby inherently match their target cluster. An example of an essential α-Keggin structure with additional atoms (non-essential structure) is shown to exemplify that *ClusterFinder* provides meaningful atomic rankings of non-essential structures. Oxygen atoms are coloured red. Atoms different from W, Fe, Mo or O are omitted for clarity.

We now use *ClusterFinder* to scan the whole ICSD for the best-fitting structure models for the experimental PDF obtained from α-Keggin clusters in solution. *ClusterFinder* uses a single unit cell of each crystal structure (188 631 structures, although we removed unreadable CIFs making it 187 469 structures) in the ICSD as the starting template. To accelerate the *ClusterFinder* process, only the scale factor was refined, and structures without W, Fe or Mo atoms (158 399 structures), or starting templates with over 1000 atoms (zero structures) were excluded. This left 29 070 candidate structures. For database screening, an isotropic contraction/expansion factor was not refined. Afterwards, the template structures from crystals in the ICSD were ranked according to their average 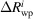 value during the *ClusterFinder* process. The complete computation took 17.5 min (1046 s) on an AMD Ryzen Threadripper 3990X with 64 cores at 2.9/4.3 GHz, or 10 h (34 882 s) on a standard laptop (Intel Core i7-8665U CPU at 1.9/2.11 GHz). Fig. 5 ▸ demonstrates that all of the top five crystal structures (Table 2 ▸) contained the α-Keggin cluster. This shows *ClusterFinder*’s ability to scan large structural databases effectively, such as the ICSD, for appropriate cluster structures.

*ClusterFinder* prioritizes starting templates exclusively comprising the essential cluster structure, *i.e.* clusters in which no atoms need removal and that thereby inherently match their target cluster, over those that contain additional atoms. Consequently, the starting template generation influences the ranking of crystal structures in the ICSD. In instances where exclusively essential clusters are present, the colour coding still reflects the internal atomic ranking, even if all atoms are good and none requires removal. Fig. 5 ▸ demonstrates this phenomenon; for instance, starting template (IV) contains only four essential α-Keggin clusters, with no atoms needing removal. However, some atoms are coloured blue, as the colour bar merely signifies the internal atomic ranking. In the case of a starting template containing essential clusters with additional atoms, as seen in Fig. 5 ▸, *ClusterFinder* indicates which atoms require removal.

**Figure 6:**
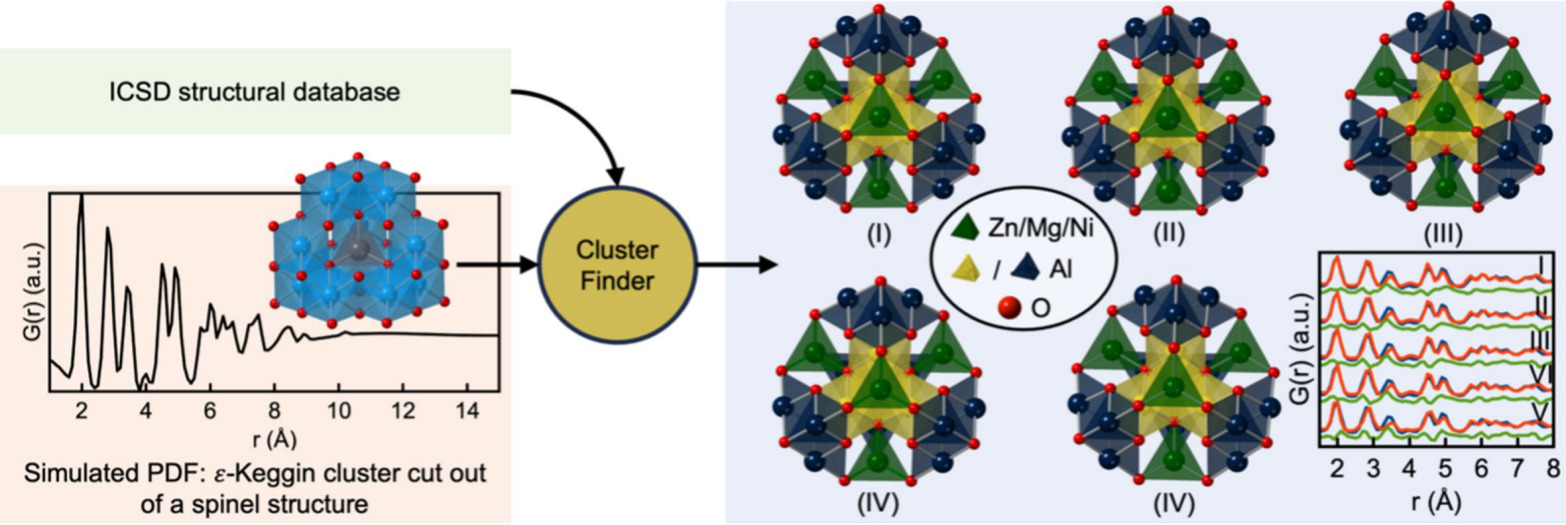
An illustration of how *ClusterFinder* is used to screen the ICSD for the correct starting model for a simulated PDF obtained from an ɛ-Keggin cluster cut out of a spinel crystal structure (coloured light blue in the left of the figure with Mg in the centre). For each structure in the ICSD, the *ClusterFinder* procedure is performed and the atoms are colour coded based on their impact on the fit quality. Afterwards, the ICSD structures are sorted according to their average 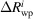 values during the *ClusterFinder* process. The five candidates with the lowest *R*_wp_ values are highlighted. More extensive views of the PDF fits, including the calculated *R*_wp_ values, can be seen in Section D in the supporting information. Atoms different from W, Fe, Mo, Al or O have been omitted for clarity.

*ClusterFinder* can also extract a cluster structure from a crystalline metal oxide structure. The ɛ-Keggin cluster serves as an excellent example of a cluster structure that can be directly cut out from a spinel structure. A PDF of an Al_12_O_40_ ɛ-Keggin cluster from the spinel MgAl_2_O_4_ crystal structure (Ji *et al.*, 2020 ▸) was simulated with parameters that mimic typical PDF dataset values, as seen in Section A in the supporting information. The PDF and structure are illustrated in Fig. 6 ▸. Again, *ClusterFinder* was used to scan all structures in the ICSD. This time, crystal structures without W, Fe, Mo or Al atoms (143 956 structures) or starting templates with more than 1000 atoms (704 structures) were excluded. After evaluation, 42 809 structures were ranked based on their average 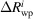 value found during the *ClusterFinder* process. The entire procedure takes 42 min (2495 s) on an AMD Ryzen Threadripper 3990X with 64 cores at 2.9/4.3 GHz or 23 h (82 100 s) on a standard laptop (Intel Core i7-8665U CPU at 1.9/2.11 GHz). The top five structures, shown in Fig. 6 ▸, are all spinel structures.

**Figure 7:**
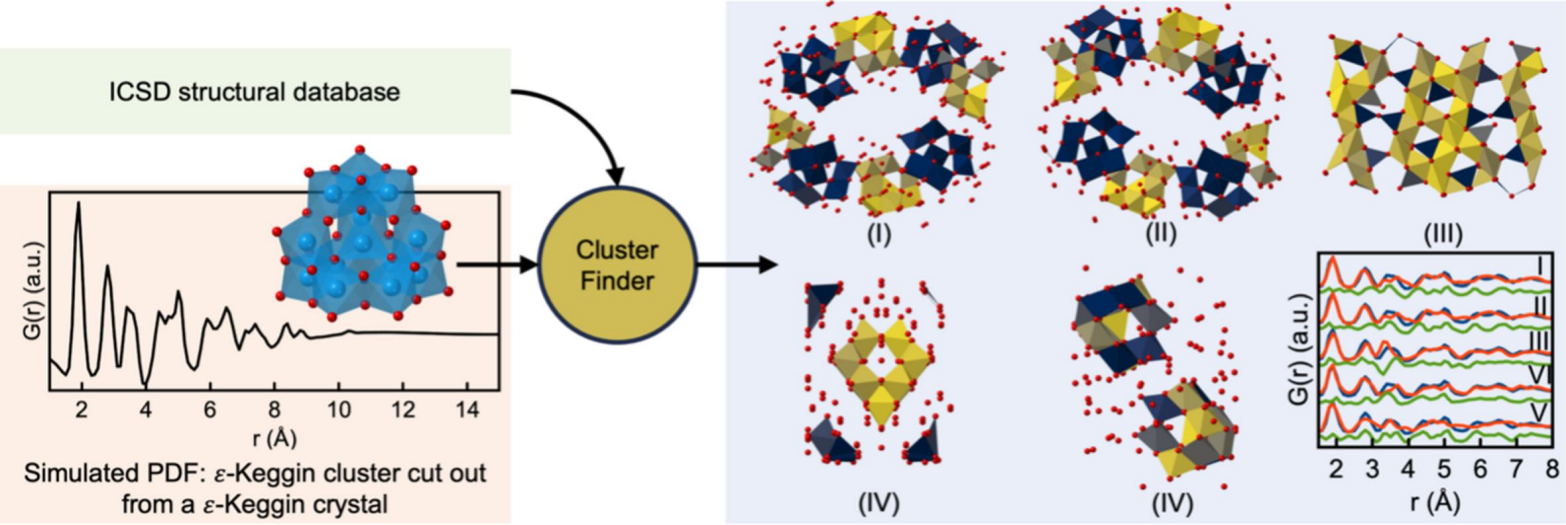
An illustration of how *ClusterFinder* is used to screen the ICSD for the correct starting model for a simulated PDF obtained from an ɛ-Keggin cluster cut out of an ɛ-Keggin crystal structure (coloured light blue in the left of the figure). For each structure in the ICSD, the *ClusterFinder* procedure is performed and the atoms are colour coded based on their impact on the fit quality. Afterwards, the ICSD structures are sorted according to their average 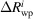 values during the *ClusterFinder* process. The five candidates with the lowest *R*_wp_ value are highlighted. More extensive views of the PDF fits, including the calculated *R*_wp_ values, can be seen in Section E in the supporting information. Oxygen atoms are coloured red. Other atoms than W, Fe, Mo, Al or O have been omitted for clarity.

We now proceed to apply *ClusterFinder* to a simulated PDF calculated from the ɛ-Keggin cluster cut out from an ɛ-Keggin crystal structure {here [Al_13_O_4_(OH)_24_(H_2_O)_12_]_2_(V_2_W_4_O_19_)_3_(OH)_2_(H_2_O)_27_; Son & Kwon, 2004 ▸} instead of a cut out from the spinel crystal structure. The ɛ-Keggin obtained in this way is more disordered than that cut out from the spinel crystal structure. The disorder can be seen in both the structures and their PDFs (Figs. 6 ▸ and 7 ▸), where the PDF simulated from the spinel-derived ɛ-Keggin (Fig. 6 ▸) exhibits sharper peaks than the PDF simulated from the ɛ-Keggin cluster cut out of the [Al_13_O_4_(OH)_24_(H_2_O)_12_]_2_(V_2_W_4_O_19_)_3_(OH)_2_(H_2_O)_27_ crystal structure (Son & Kwon, 2004 ▸) (Fig. 7 ▸). Again, we use *Cluster­Finder* on all ICSD structures containing W, Fe, Mo or Al atoms one by one. Afterwards, it ranks the structures based on their average 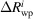 value obtained during the *Cluster­Finder* process. Fig. 7 ▸ and Table 4 show that the top five structures mainly contain ɛ-Keggin clusters or are variants of the spinel structure [structures (III) and (V)]. While α-Keggin and ɛ-Keggin clusters are very similar and only distinct in the different rotational orientations of their four *M*_3_O_13_ units, *ClusterFinder* is able to differentiate between them in starting template structures (I) and (II) where the α-Keggin motif is removed (blue) and the ɛ-Keggin motifs are kept (yellow).

*ClusterFinder* can, moreover, discern between the more ordered spinel-obtained motifs (Fig. 6 ▸ and Table 3 ▸) and the more distorted Keggin crystal structure (Fig. 7 ▸ and Table 4 ▸), which demonstrates that it is sensitive to minor changes in the PDF. This highlights the level of detailed description attained in this modelling approach.

In Sections F and G in the supporting information, we present two similar examples in which we rank the ICSD structures according to experimental datasets obtained from ionic [Bi_38_O_45_] clusters and ceria (CeO_2_) nanoparticles. We find that the highest ranked structures from the [Bi_38_O_45_] cluster example are δ-Bi_2_O_3_ crystal structures, as previously observed by Weber *et al.* (2017 ▸). For the ceria nanoparticles, the highest ranked structures correspond to bixbyite-type structures, which are related to the fluorite-type structure that CeO_2_ would be expected to take. This demonstrates that, while *ClusterFinder* often provides results closely related to the true chemical solution, validation and considerations of structure relations are still required in the data analysis process.

## 4. Conclusions

We have introduced a new automated structure selection approach called *ClusterFinder* for identifying suitable starting models for analysis and refinement of PDFs from nano­clusters. The premise of *ClusterFinder* is that the structure of a nanocluster can probably be described as a fragment of an already published crystal structure, and it thus screens crystal structures and identifies fragments for further analysis. The structure found by *ClusterFinder* is not necessarily a unique solution to the PDF, but *ClusterFinder*’s automated process ensures a systematic and extensive screening of a range of possible structures.

*ClusterFinder* is inspired by our previously developed algorithms, *LIGA* and *ML-MotEx*, but is significantly faster, facilitating screening of large databases for cluster identification in minutes. Our study demonstrates *ClusterFinder*’s efficacy as a robust tool for extracting appropriate starting models from extensive structural databases like the ICSD. By applying *ClusterFinder* to PDFs from various nanoclusters, such as α-Keggin clusters, ɛ-Keggin clusters, ionic [Bi_38_O_45_] clusters and ceria nanoparticles, we have showcased its abilities in effectively ranking and selecting the most relevant structure models based on fit quality.

All the data supporting this study are available either within the paper, as supporting information or on the associated GitHub to the paper, https://github.com/AndySAnker/ClusterFinder. The code supporting this study is also available on the same associated GitHub.

## 5. Related literature

For further literature related to the supporting information, see Anker *et al.* (2021 ▸), Artini *et al.* (2014 ▸), Chakraborty *et al.* (2006 ▸), Coduri *et al.* (2013 ▸), Estes *et al.* (2016 ▸), Juhás *et al.* (2013 ▸), Labidi *et al.* (2008 ▸), Rademacher *et al.* (2001 ▸), Radosavljević-Evans *et al.* (2002 ▸), Sasaki *et al.* (2004 ▸) and Yang *et al.* (2014 ▸).

## Supplementary Material

Additional background. DOI: 10.1107/S2053273324001116/tw5008sup1.pdf

## Figures and Tables

**Table 1:** Four starting models containing the α-Keggin clusters used with *Cluster­Finder* to extract an α-Keggin cluster

Starting model	Crystal composition	Reference
1	[Hpy]_4_H_2_[H_2_W_12_O_40_] (py = pyridine)	Niu *et al.* (2004 ▸)
2	[(CH_3_)_4_N]_4_SiW_12_O_40_	Joachim *et al.* (1981 ▸)
3	([(CH_3_)_2_NH_2_]_6_{Cu[HCON(CH_3_)_2_]_4_}(GeW_12_O_40_)_2_)[HCON(CH_3_)_2_]_2_	Niu *et al.* (2003 ▸)
4	[(CH_3_)_2_NH_2_]_3_(PW_12_O_40_)	Busbongthong & Ozeki (2009 ▸)

**Table 2:** Crystal composition of the top five candidate crystal structures ranked by *ClusterFinder* for the PDF obtained from α-Keggin clusters in solution

Ranked structure	Crystal composition	Reference
(I)	[(CH_3_)_4_N]_6_[Cu_0.5_(H_2_)_0.5_O_4_W_12_O_36_](H_2_O)_10_	Lunk *et al.* (1993 ▸)
(II)	Cs_5_[Cr_3_O(OOCH)_6_(H_2_O)_3_](CoW_12_O_40_)(H_2_O)_2_	Uchida *et al.* (2006 ▸)
(III)	[(CH_3_)_4_N]_6_(H_2_W_12_O_40_)(H_2_O)_9_	Asami *et al.* (1984 ▸)
(IV)	[Al_13_O_4_(OH)_24_(H_2_O)_12_](H_2_W_12_O_40_)(OH)(H_2_O)_23.12_	Son *et al.* (2003 ▸)
(V)	K_2_(H_2_O)_4_Eu(H_2_O)_7_[Eu(H_2_O)_3_HAlW_11_O_39_](H_2_O)_7_	Niu *et al.* (2013 ▸)

**Table 3:** Crystal composition of the top five candidate crystal structures ranked by *ClusterFinder* for the simulated PDF from the Al_12_O_40_ ɛ-Keggin cluster cut out from the spinel MgAl_2_O_4_ crystal structure

Ranked structure	Crystal composition	Reference
(I)	NiAl_2_O_4_	Vegard & Borlaug (1943 ▸)
(II)	MgAl_2_O_4_	Zorina & Kvitka (1968 ▸)
(III)	ZnAl_2_O_4_	Holgersson (1927 ▸)
(IV)	ZnAl_2_O_4_	Vegard & Borlaug (1943 ▸)
(V)	ZnAl_2_O_4_	Saalfeld (1964 ▸)

**Table 4:** Crystal composition of the top five candidate crystal structures calculated by *ClusterFinder* for the simulated PDF from the ɛ-Keggin cluster cut out of the Al_12_O_40_ [Al_13_O_4_(OH)_24_(H_2_O)_12_]_2_(V_2_W_4_O_19_)_3_(OH)_2_(H_2_O)_27_ crystal structure (Son & Kwon, 2004 ▸)

Ranked structure	Crystal composition	Reference
(I)	[Al_13_O_4_(OH)_24_(H_2_O)_12_](H_2_W_12_O_40_)(OH)(H_2_O)_23.12_	Son *et al.* (2003 ▸)
(II)	[Al_13_O_4_(OH)_24_(H_2_O)_12_](CoW_12_O_40_)(OH)(H_2_O)_20_	Son *et al.* (2003 ▸)
(III)	Ca_2_Mg_2_Fe_2_[Al_14_O_31_(OH)](Al_2_O)(Al)[Al(OH)]	Rastsvetaeva *et al.* (2010 ▸)
(IV)	[(GeO_4_)Al_12_(OH)_24_(H_2_O)_12_](SeO_4_)_4_(H_2_O)_14_	Lee *et al.* (2001 ▸)
(V)	(Al_2_O_3_)_13_(SO_3_)_6_(H_2_O)_79_	Nordstrom (1982 ▸)

## References

[bb50] Anker, A. S., Christiansen, T. L., Weber, M., Schmiele, M., Brok, E., Kjær, E. T. S., Juhás, P., Thomas, R., Mehring, M. & Jensen, K. M. Ø. (2021). *Angew. Chem. Int. Ed.* **60**, 2–12. 10.1002/anie.202103641 PMC8456784 34056798

[bb1] Anker, A. S., Kjær, E. T. S., Dam, E. B., Billinge, S. J. L., Jensen, K. M. Ø. & Selvan, R. (2020). In *Proceedings of the 16th International Workshop on Mining and Learning with Graphs (MLG)*, 24 August 2020, San Diego, California, USA (virtual). New York: Association for Computing Machinery. https://www.mlgworkshop.org/2020/.

[bb2] Anker, A. S., Kjær, E. T. S., Juelsholt, M., Christiansen, T. L., Skjærvø, S. L., Jørgensen, M. R. V., Kantor, I., Sørensen, D. R., Billinge, S. J. L., Selvan, R. & Jensen, K. M. Ø. (2022). *NPJ Comput. Mater.* **8**, 213.

[bb3] Anker, A. S., Kjær, E. T. S., Juelsholt, M. & Jensen, K. M. Ø. (2024). *J. Appl. Cryst.* **57**, 34–43. 10.1107/S1600576723010014 PMC10840315 38322723

[bb51] Artini, C., Pani, M., Lausi, A., Masini, R. & Costa, G. A. (2014). *Inorg. Chem.* **53**, 10140–10149. 10.1021/ic5011242 25192043

[bb4] Asami, M., Ichida, H. & Sasaki, Y. (1984). *Acta Cryst.* C**40**, 35–37.

[bb5] Banerjee, S., Liu, C.-H., Jensen, K. M. Ø., Juhás, P., Lee, J. D., Tofanelli, M., Ackerson, C. J., Murray, C. B. & Billinge, S. J. L. (2020). *Acta Cryst.* A**76**, 24–31. 10.1107/S2053273319013214 PMC7045905 31908346

[bb6] Billinge, S. J. L. & Levin, I. (2007). *Science*, **316**, 561–565. 10.1126/science.1135080 17463280

[bb7] Busbongthong, S. & Ozeki, T. (2009). *Bull. Chem. Soc. Jpn*, **82**, 1393–1397.

[bb8] Castillo-Blas, C., Moreno, J. M., Romero-Muñiz, I. & Platero-Prats, A. E. (2020). *Nanoscale*, **12**, 15577–15587. 10.1039/d0nr01673j 32510095

[bb52] Chakraborty, K. R., Krishna, P. S. R., Chavan, S. V. & Tyagi, A. K. (2006). *Powder Diffr.* **21**, 36–39.

[bb9] Chen, X. & Yamanaka, S. (2002). *Chem. Phys. Lett.* **360**, 501–508.

[bb10] Christiansen, T. L., Cooper, S. R. & Jensen, K. M. Ø. (2020). *Nanoscale Adv.* **2**, 2234–2254. 10.1039/d0na00120a PMC9418950 36133369

[bb11] Cliffe, M. J., Dove, M. T., Drabold, D. & Goodwin, A. L. (2010). *Phys. Rev. Lett.* **104**, 125501. 10.1103/PhysRevLett.104.125501 20366543

[bb12] Cliffe, M. J. & Goodwin, A. L. (2013). *J. Phys. Condens. Matter*, **25**, 454218. 10.1088/0953-8984/25/45/454218 24140797

[bb53] Coduri, M., Scavini, M., Allieta, M., Brunelli, M. & Ferrero, C. (2013). *Chem. Mater.* **25**, 4278–4289.

[bb13] Du, P., Kokhan, O., Chapman, K. W., Chupas, P. J. & Tiede, D. M. (2012). *J. Am. Chem. Soc.* **134**, 11096–11099. 10.1021/ja303826a 22720737

[bb14] Egami, T. & Billinge, S. J. L. (2012). *Underneath the Bragg Peaks.* Oxford: Pergamon.

[bb54] Estes, S. L., Antonio, M. R. & Soderholm, L. (2016). *J. Phys. Chem. C*, **120**, 5810–5818.

[bb15] Holgersson, S. (1927). *Lunds Universitets Årsskrift. NF Avd.* **2**, 1–9.

[bb16] Ji, H., Hou, X., Molokeev, M. S., Ueda, J., Tanabe, S., Brik, M. G., Zhang, Z., Wang, Y. & Chen, D. (2020). *Dalton Trans.* **49**, 5711–5721. 10.1039/d0dt00931h 32297895

[bb17] Joachim, F., Axel, T. & Rosemarie, P. (1981). *Z. Naturforsch.* **36**, 161–171.

[bb18] Juelsholt, M., Lindahl Christiansen, T. & Jensen, K. M. Ø. (2019). *J. Phys. Chem. C*, **123**, 5110–5119.

[bb19] Juhás, P., Cherba, D. M., Duxbury, P. M., Punch, W. F. & Billinge, S. J. L. (2006). *Nature*, **440**, 655–658. 10.1038/nature04556 16572167

[bb55] Juhás, P., Davis, T., Farrow, C. L. & Billinge, S. J. L. (2013). *J. Appl. Cryst.* **46**, 560–566.

[bb20] Juhás, P., Farrow, C., Yang, X., Knox, K. & Billinge, S. (2015). *Acta Cryst.* A**71**, 562–568. 10.1107/S2053273315014473 26522405

[bb21] Juhás, P., Granlund, L., Duxbury, P. M., Punch, W. F. & Billinge, S. J. L. (2008). *Acta Cryst.* A**64**, 631–640. 10.1107/S0108767308027591 18931419

[bb22] Juhás, P., Granlund, L., Gujarathi, S. R., Duxbury, P. M. & Billinge, S. J. L. (2010). *J. Appl. Cryst.* **43**, 623–629.

[bb23] Kjær, E. T. S., Anker, A. S., Weng, M. N., Billinge, S. J. L., Selvan, R. & Jensen, K. M. Ø. (2023). *Digit. Discov.* **2**, 69–80. 10.1039/d2dd00086e PMC9923795 36798882

[bb24] Kløve, M., Sommer, S., Iversen, B. B., Hammer, B. & Dononelli, W. (2023). *Adv. Mater.* **35**, 2208220. 10.1002/adma.202208220 36630711

[bb56] Labidi, O., Drache, M., Roussel, P. & Wignacourt, J.-P. (2008). *Solid State Sci.* **10**, 1074–1082.

[bb25] Lee, A. P., Phillips, B. L., Olmstead, M. M. & Casey, W. H. (2001). *Inorg. Chem.* **40**, 4485–4487. 10.1021/ic010146e 11487360

[bb27] Lunk, H.-J., Giese, S., Fuchs, J. & Stösser, R. (1993). *Z. Anorg. Allg. Chem.* **619**, 961–968.

[bb28] Magnard, N. P. L., Anker, A. S., Aalling-Frederiksen, O., Kirsch, A. & Jensen, K. M. Ø. (2022). *Dalton Trans.* **51**, 17150–17161. 10.1039/d2dt02153f PMC9678240 36156665

[bb29] Niu, J., Zhao, J., Wang, J. & Bo, Y. (2004). *J. Coord. Chem.* **57**, 935–946.

[bb30] Niu, J.-Y., Han, Q.-X. & Wang, J.-P. (2003). *J. Coord. Chem.* **56**, 523–530.

[bb31] Niu, L., Li, Z., Xu, Y., Sun, J., Hong, W., Liu, X., Wang, J. & Yang, S. (2013). *Appl. Mater. Interfaces*, **5**, 8044–8052. 10.1021/am402127u 23910723

[bb32] Nordstrom, D. K. (1982). *Geochim. Cosmochim. Acta*, **46**, 681–692.

[bb33] Poimanova, O. Y., Radio, S. V., Bilousova, K. Y., Baumer, V. N. & Rozantsev, G. M. (2015). *J. Coord. Chem.* **68**, 1–17.

[bb34] Proffen, Th. & Neder, R. B. (1997). *J. Appl. Cryst.* **30**, 171–175.

[bb35] Proffen, Th. & Neder, R. B. (1999). *J. Appl. Cryst.* **32**, 838–839.

[bb57] Rademacher, O., Göbel, H., Ruck, M. & Oppermann, H. (2001). *Z. Kristallogr. New Cryst. Struct.* **216**, 29–30.

[bb58] Radosavljevic Evans, I., Tao, S., Irvine, J. T. S. & Howard, J. A. K. (2002). *Chem. Mater.* **14**, 3700–3704.

[bb36] Rastsvetaeva, R., Aksenov, S. & Verin, I. (2010). *Crystallogr. Rep.* **55**, 563–568.

[bb37] Redrup, K. V. & Weller, M. T. (2009). *Dalton Trans.* pp. 4468–4472. 10.1039/b818103a 19488444

[bb38] Saalfeld, H. (1964). *Z. Kristallogr. Cryst. Mater.* **120**, 476–478.

[bb59] Sasaki, T., Ukyo, Y., Kuroda, K., Arai, S., Muto, S. & Saka, H. (2004). *J. Ceram. Soc. Jpn*, **112**, 440–444.

[bb39] Son, J.-H. & Kwon, Y.-U. (2004). *Inorg. Chem.* **43**, 1929–1932. 10.1021/ic035278h 15018512

[bb40] Son, J. H., Kwon, Y.-U. & Han, O. H. (2003). *Inorg. Chem.* **42**, 4153–4159. 10.1021/ic0340377 12817975

[bb41] Uchida, S., Kawamoto, R. & Mizuno, N. (2006). *Inorg. Chem.* **45**, 5136–5144. 10.1021/ic060684x 16780336

[bb42] Vegard, L. & Borlaug, A. (1943). *Avhandlinger/Norske Videnskaps-Akademi, Matematisk-Naturvidenskapelig Klasse*. Oslo: Dybwad [in Komm.].

[bb43] Weber, M., Schlesinger, M., Walther, M., Zahn, D., Schalley, C. A. & Mehring, M. (2017). *Z. Kristallogr. Cryst. Mater.* **232**, 185–207.

[bb44] Yang, L., Juhás, P., Terban, M. W., Tucker, M. G. & Billinge, S. J. L. (2020). *Acta Cryst.* A**76**, 395–409. 10.1107/S2053273320002028 PMC7233026 32356790

[bb60] Yang, X., Juhas, P., Farrow, C. L. & Billinge, S. J. (2014). arXiv:1402.3163.

[bb45] Zagorac, D., Müller, H., Ruehl, S., Zagorac, J. & Rehme, S. (2019). *J. Appl. Cryst.* **52**, 918–925. 10.1107/S160057671900997X PMC6782081 31636516

[bb46] Zorina, N. & Kvitka, S. (1968). *Kristallografiya*, **13**, 703–705.

