# Supplementary material for: ClusterFinder: a fast tool to find cluster structures from pair distribution function data
Source: Acta Crystallogr A Found Adv. 2024 Feb 29;80(Pt 2):213–20. doi: 10.1107/S2053273324001116 (PMC10913672; doi:10.1107/S2053273324001116)
Supplement: Supplementary file 1 [file a-80-00213-sup1.pdf]

Acta Cryst A
FOUNDATIONS
ADVANCES

Volume 80 (2024)

Supporting information for article:

ClusterFinder: a fast tool to find cluster structures from pair distribution function data

Andy S. Anker, Ulrik Friis-Jensen, Frederik L. Johansen, Simon J. L Billinge and Kirsten M. Ø. Jensen

Table of Contents

A: PDF calculation parameters for all the examples provided in the paper.. 3

B: Meaning of the atom colour coding after the ClusterFinder process. 3

C: ClusterFinder analysis on the simulated PDFs from Figure 3... 3

D: Fitting the five best candidates from the ClusterFinder process to simulated PDF obtained from a ε-keggin cluster cut out of a spinel crystal 5

E: Fitting the five best candidates from the ClusterFinder process to simulated PDF obtained from a ε-keggin cluster cut out of an ε-keggin crystal 6

F: Using ClusterFinder to screen the ICSD for the correct starting model of an experimental PDF obtained from a [$Bi_{38}O_{45}$] cluster in solution... 6

G: Using ClusterFinder to screen the ICSD for the correct starting model of an experimental PDF obtained from ceria nanoparticles... 8

H: Experimental details of the ceria example...10

A: PDF calculation parameters for all the examples provided in the paper

	**Figure 1:** Simulated PDFs	**Figure 2:** A single C_{60} buckyball	**Figure 2:** Simulated paratungstate and decatungstate clusters	**Figure 3+4:** A $W_{12}O_{40}$ unit (data collected at DanMax)
r-range (Å)	0 – 30.1	3 – 12	0 – 30.1	1.6 – 10
r-step	0.01	0.01	0.01	0.01
Q_{min} (Å^{-1})	0.7	0.5	0.7	0.7
Q_{max} (Å^{-1})	25	24	25	20
Q_{damp} (Å^{-1})	0.04	0.04	0.04	0.05
ADP (Å^2)	0.3	0.3	0.3	0.3

Table 1 | PDF calculation parameters for all the examples provided in the paper.

B: Meaning of the atom colour coding after the ClusterFinder process.

Effect on the R_{wp} when the atom is present in the structure	ΔR^i_{wp}	Denomination of the atom	Colour code
Negative (-)	Negative (-)	Good atom	Yellow
Positive (+)	Positive (+)	Bad atom	Blue

Table 2. Meaning of the atom colour coding after the ClusterFinder process.

C: ClusterFinder analysis on the simulated PDFs from Figure 3

Structural templates illustrated in Figure 3 are made by creating:

- B / Blue: a 2x2x2 supercell from the $Na_5(H_7W_{12}O_{42})(H_2O)_{20}$ crystal structure (Redrup & Weller, 2009) which contains 1216 atoms;
- C / Green: a single unit cell of a C_{60} crystal structure (Chen & Yamanaka, 2002) which contains 132 atoms.
- D / Red: a 1x1x2 supercell from the $(Ba(H_2O)_2(H(N(CH_3)_2)CO)_3)_2(W_{10}O_{32})(H(N(CH_3)_2)CO)_2$ crystalline model (Poimanova *et al.*, 2015) which contains 160 atoms.

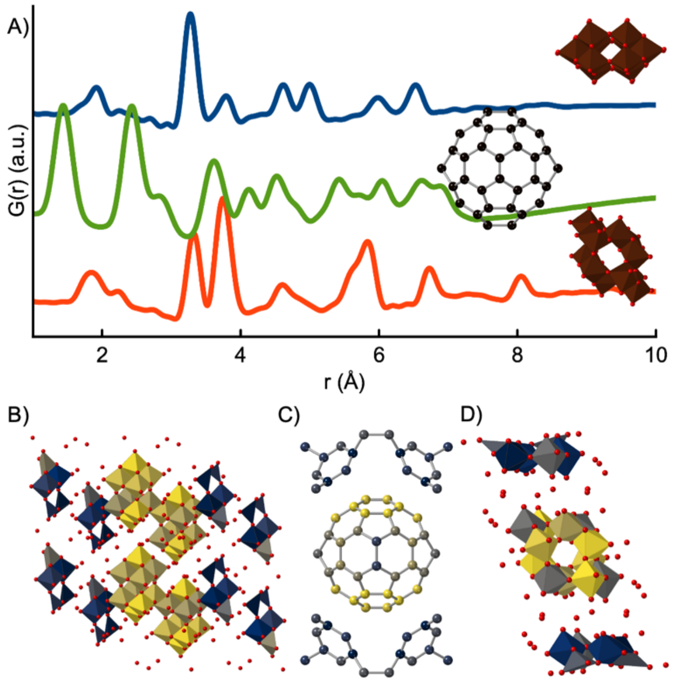

Fig. S1 | Analysis of simulated PDFs from well-known cluster structures. A) Simulated PDFs of blue) a decatungstate polyoxometalate cluster cut out the $Na_5(H_7W_{12}O_{42})(H_2O)_{20}$ crystal (Redrup & Weller, 2009), green) a C_{60} buckyball cut out from a single unit cell of a crystal structure of C_{60} (Chen & Yamanaka, 2002), and red) a paratungstate polyoxometalate cluster cut out of the $(Ba(H_2O)_2(H(N(CH_3)_2)CO)_3)_2(W_{10}O_{32})(H(N(CH_3)_2)CO)_2$ crystal (Poimanova *et al.*, 2015). The simulation parameters mimic typical values of a PDF dataset and can be seen in Table S1. B-D) Results of using ClusterFinder on the three simulated PDFs colouring the atom contribution values with a continuous colour bar. Oxygens are coloured red and polyhedra are coloured according to their metal atom center.

D: Fitting the five best candidates from the ClusterFinder process to simulated PDF obtained from a ε-keggin cluster cut out of a spinel crystal

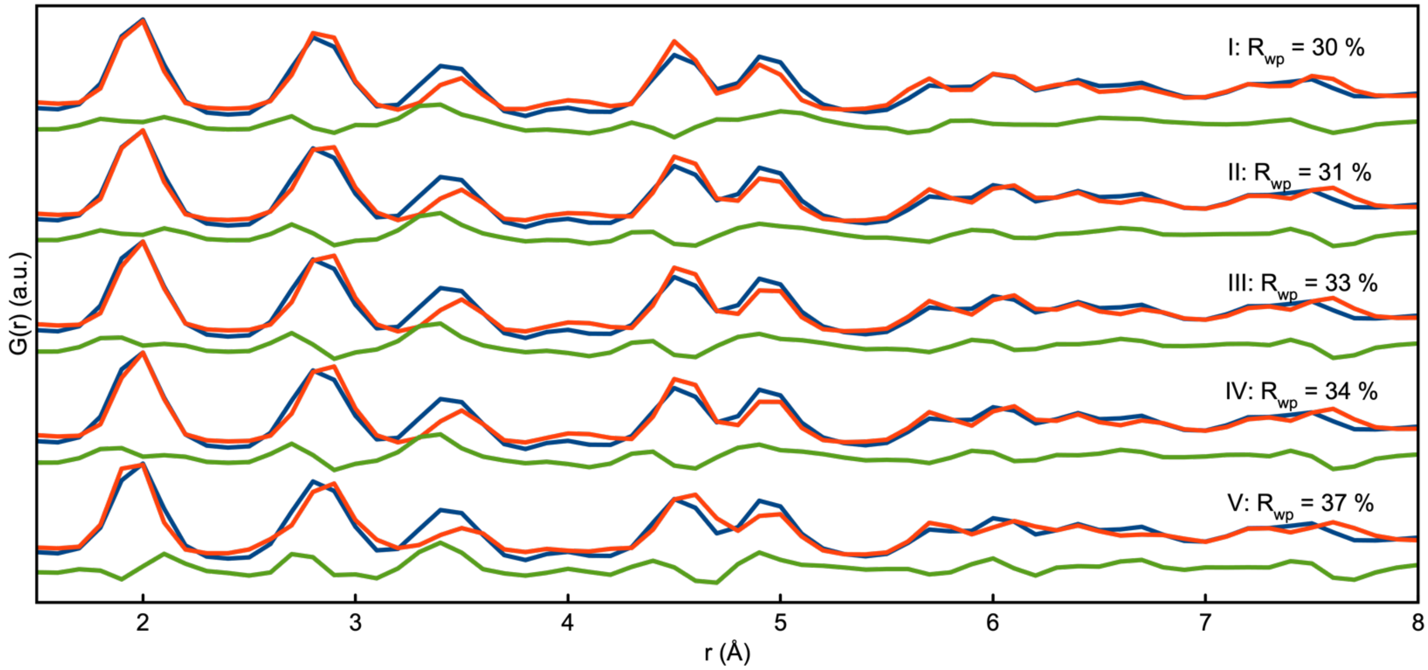

Fig. S2 | PDF fits of the top five structures from the ClusterFinder process used to screen ICSD for the correct starting model of a simulated PDF obtained from a ε-keggin cluster cut out of a spinel crystal.

E: Fitting the five best candidates from the ClusterFinder process to simulated PDF obtained from a ε-keggin cluster cut out of an ε-keggin crystal

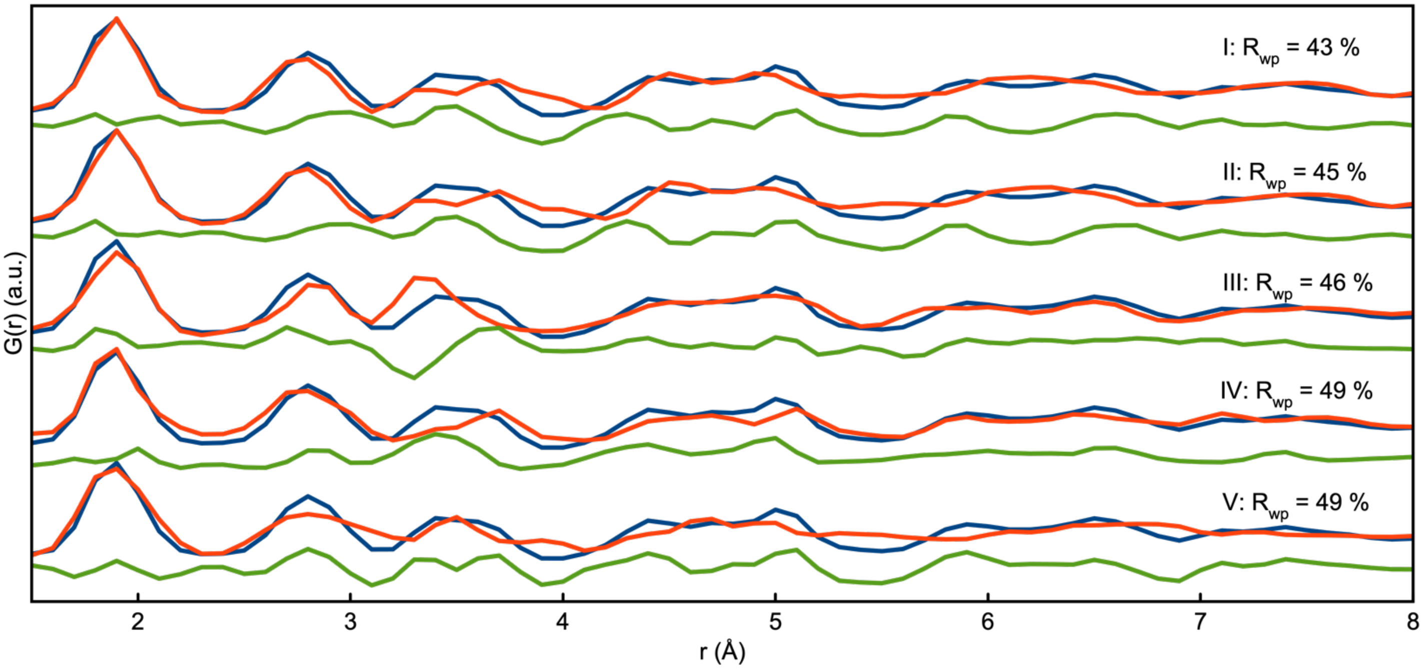

Fig. S3 | PDF fits of the top five structures from the ClusterFinder process used to screen ICSD for the correct starting model of a simulated PDF obtained from a ε-keggin cluster cut out of an ε-keggin crystal.

F: Using ClusterFinder to screen the ICSD for the correct starting model of an experimental PDF obtained from a [$Bi_{38}O_{45}$] cluster in solution

We use ClusterFinder to scan a database of the best-fitting structure models from the experimental PDF of [$Bi_{38}O_{45}$] clusters in solution (Anker *et al.*, 2021). The [$Bi_{38}O_{45}$] type clusters are previously seen to be highly similar to cut-outs of the β-Bi_2O_3 and the δ-Bi_2O_3 crystal structures (Weber *et al.*, 2017).

Again, we use ClusterFinder iteratively on each structure (188,631 structures) in the ICSD. To accelerate ClusterFinder, we only refine the scale factor, and we remove all crystals not containing Bi atoms (181,215 structures) and supercells with more than 1000 atoms (704 structures), leaving us with 5550 structures. Afterwards, we rank the structures in ICSD by the average R_{wp} value during the ClusterFinder process. The entire procedure takes 8 min (468 seconds) on an AMD Ryzen Threadripper 3990X with 64-core 2.9/4.3GHz and 95

min (5727 s) on an Intel(R) Core™ i7-8665U CPU @ 1.9/2.11 GHz. Figure S4 demonstrates that the crystal structures in the top five are mainly fluorite δ-Bi_2O_3 type structures or similar:

1) $Cd_{3.573}Bi_{19.427}V_4O_{42}$,(Labidi *et al.*, 2008) 2) $Ca_{4.207}Bi_{18.793}V_4O_{42}$,(Labidi *et al.*, 2008) 3) $Bi_{4.66}Ca_{1.09}VO_{10.5}$ (Radosavljevic Evans *et al.*, 2002), and 5) $Na_{1.533}Bi_{21.467}V_4O_{42}$ (Labidi *et al.*, 2008), but the 4th ranked structure is a Bi_2SeO_5 crystal instead (Rademacher *et al.*, 2001).

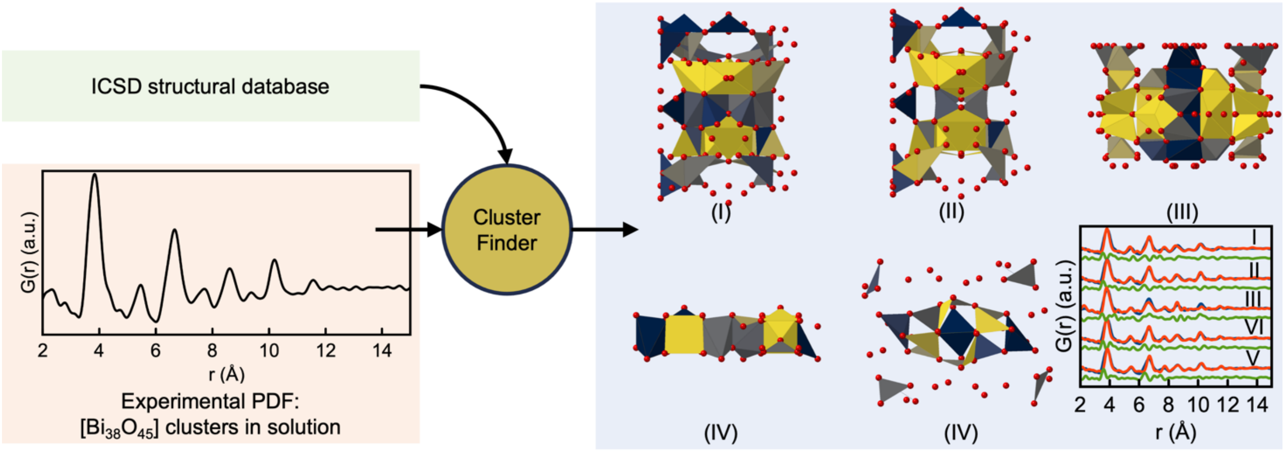

Fig. S4 | Illustration of how ClusterFinder is used to screen ICSD for the correct starting model of an experimental PDF obtained from a [$Bi_{38}O_{45}$] cluster in solution. For each structure in the ICSD, the ClusterFinder procedure is performed, and the atoms are coloured according to their impact on the fit quality using a continuous colour bar. The structures in ICSD are afterwards sorted after their average R_{wp} value during the ClusterFinder procedure. The five candidates with the lowest R_{wp} value are highlighted along with their fits to the dataset, only fitting the scaling factor. More extensive PDF fits, including R_{wp} values, can be seen in Figure S5. Oxygen atoms are coloured red. Other atoms than Bi and O are omitted for clarity.

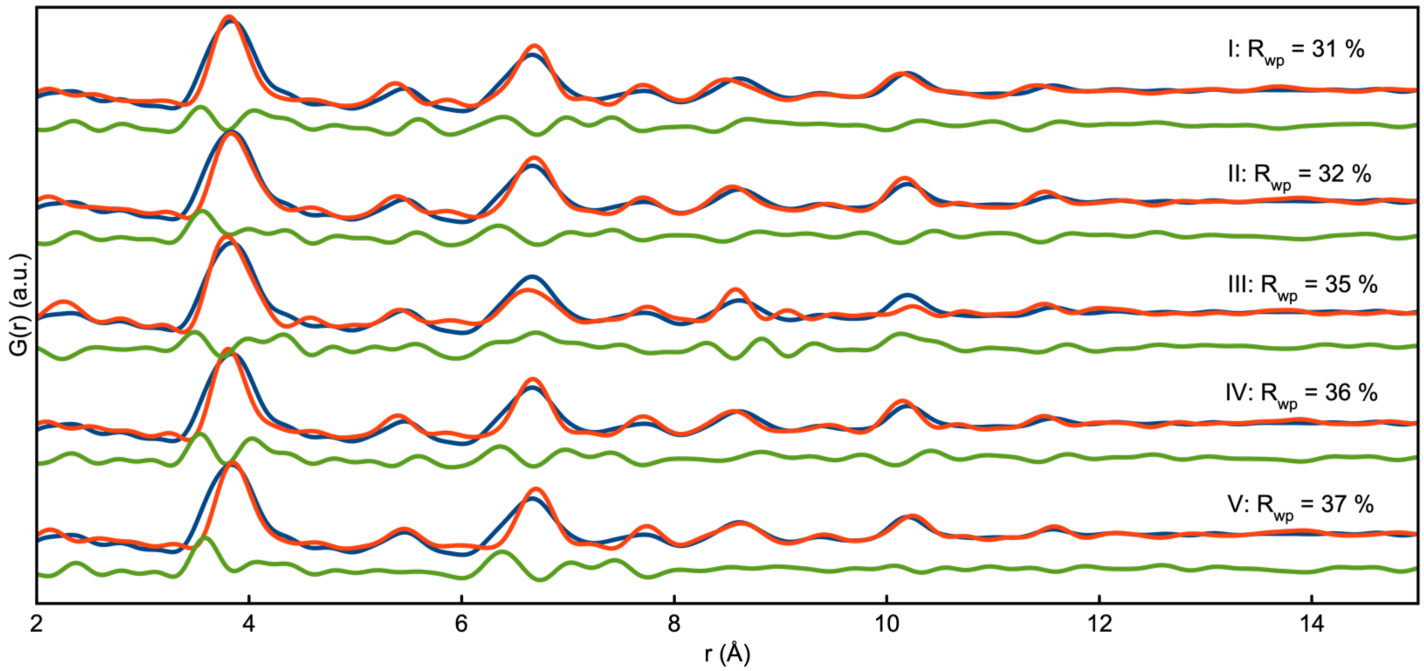

Fig. S5 | PDF fits of the top five structures from the ClusterFinder process used to screen ICSD for the correct starting model of a simulated PDF obtained from a [$Bi_{38}O_{45}$] cluster in solution.

G: Using ClusterFinder to screen the ICSD for the correct starting model of an experimental PDF obtained from ceria nanoparticles

We use ClusterFinder to scan a database of the best-fitting structure models from the experimental PDF of a ceria nanoparticle. Experimental details are provided in section H. Iteratively, we use ClusterFinder on each structure (188,631 structures) in the ICSD. To accelerate ClusterFinder, we only refine the scale factor, and we remove crystals not containing Ce atoms (181,311 structures) and supercells with more than 1000 atoms (704 structures), leaving us with 5454 structures. Afterwards, we rank the structures in ICSD by the average R_{wp} value during the ClusterFinder process. The entire procedure takes 3 min (183 s) on an AMD Ryzen Threadripper 3990X with 64-core 2.9/4.3GHz and 56 min (3342 s) on an Intel(R) Core™ i7-8665U CPU @ 1.9/2.11 GHz. Figure S6 demonstrates that all crystal structures in the top 4 are bixbyite-type structures, which are closely related to the fluorite-type structure:

1) $Ce_{0.6875}Y_{0.3125}O_2$ (Coduri *et al.*, 2013), 2) $Ce_{0.65625}Y_{0.34375}O_2$ (Coduri *et al.*, 2013), 3) $Ce_{0.9}Gd_{0.1}O_{1.95}$ (Artini *et al.*, 2014), and 4) $Ce_{0.5}Nd_{0.5}O_{1.75}$ (Chakraborty *et al.*, 2006), whereas the 5th ranked structure is a 5) $Ce_2Zr_2O_7$ pyrochlore structure (Sasaki *et al.*, 2004).

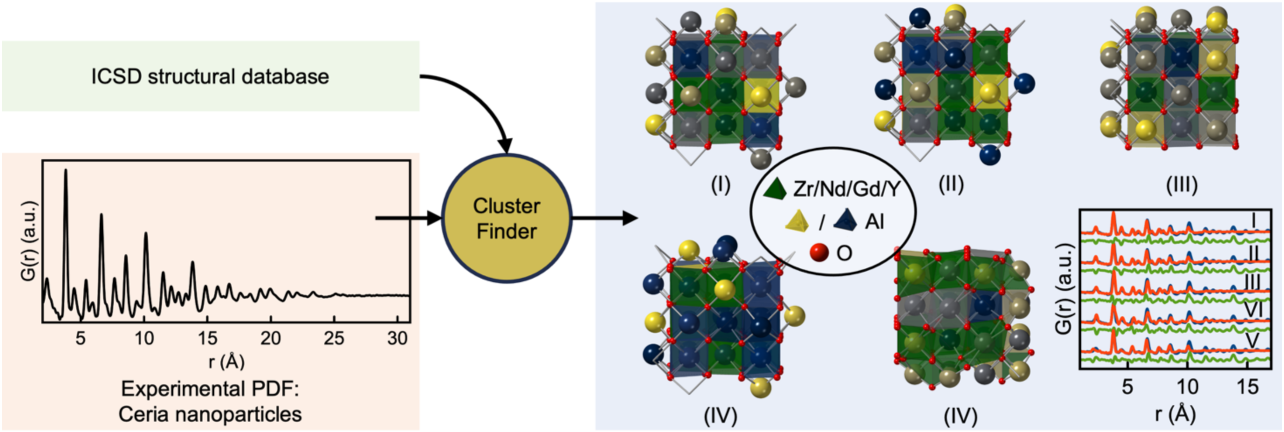

Fig. S6 | Illustration of how ClusterFinder is used to screen ICSD for the correct starting model of an experimental PDF obtained from ceria nanoparticles. For each structure in the ICSD, the ClusterFinder procedure is performed, and the atoms are coloured according to their impact on the fit quality using a continuous colour bar. The structures in ICSD are afterwards sorted after their average R_{wp} value during the ClusterFinder procedure. The five candidates with the lowest R_{wp} value are highlighted along with their fits to the dataset, only fitting the scaling factor. More extensive PDF fits, including R_{wp} values, can be seen in Figure S7. Oxygen atoms are coloured red. Other atoms than Ce and O are omitted for clarity.

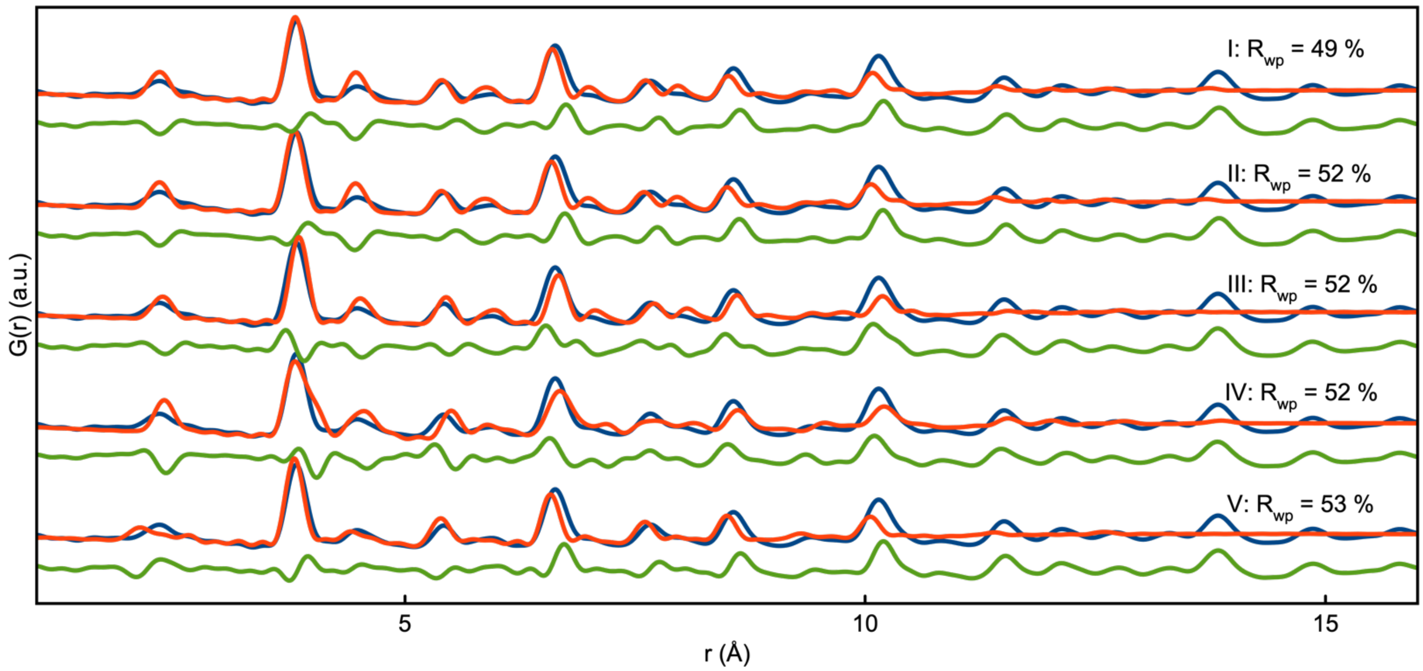

Fig. S7 | PDF fits of the top five structures from the ClusterFinder process used to screen ICSD for the correct starting model of a simulated PDF obtained from ceria nanoparticles.

H: Experimental details of the ceria example

Synthesis of 2.5 nm CeO_2 nanoparticles:

500 mg $[Ce_6(\mu_3\text{-}O)_4(\mu_3\text{-}OH)_4(NH_3CH_2COO)_8\text{-}(NO_3)_4(H_2O)_6]Cl_8{\cdot}8H_2O$ crystals (synthesised following the procedure described by Estes et al. (Estes *et al.*, 2016)) were dissolved in 10 mL DMSO at 80°C until fully dissolved. Afterwards, 10 mL (0.5 M) NaOH was added while stirring vigorously for 3 minutes. The amorphous powder was then washed three times with demineralised water and annealed at 60°C for 3 hrs.

Total Scattering Data:

The powder was transferred to a Kapton tube with an inner diameter of 1.05 mm and X-ray total scattering data were collected using the RA-PDF geometry with x-ray wavelength 0.2072 Å at beamline P02.1 at PETRAIII, DESY, Hamburg. The data were integrated using Fit2D, (Yang *et al.*, 2014) and PDFs were obtained using PDFgetx3. (Juhas *et al.*, 2013)

References

Anker, A. S., Christiansen, T. L., Weber, M., Schmiele, M., Brok, E., Kjær, E. T. S., Juhás, P., Thomas, R., Mehring, M. & Jensen, K. M. Ø. (2021). *Angew. Chem. Int. Ed.* **60**, 2-12.

Artini, C., Pani, M., Lausi, A., Masini, R. & Costa, G. A. (2014). *Inorg. Chem.* **53**, 10140-10149.

Chakraborty, K. R., Krishna, P. S. R., Chavan, S. V. & Tyagi, A. K. (2006). *Powder Diffr.* **21**, 36-39.

Chen, X. & Yamanaka, S. (2002). *Chem. Phys. Lett.* **360**, 501-508.

Coduri, M., Scavini, M., Allieta, M., Brunelli, M. & Ferrero, C. (2013). *Chem. Mater.* **25**, 4278-4289.

Estes, S. L., Antonio, M. R. & Soderholm, L. (2016). *J. Phys. Chem. C* **120**, 5810-5818.

Juhas, P., Davis, T., Farrow, C. L. & Billinge, S. J. L. (2013). *J. Appl. Cryst.* **46**, 560-566.

Labidi, O., Drache, M., Roussel, P. & Wignacourt, J.-P. (2008). *Solid State Sci.* **10**, 1074-1082.

Poimanova, O. Y., Radio, S. V., Bilousova, K. Y., Baumer, V. N. & Rozantsev, G. M. (2015). *J. Coord. Chem.* **68**, 1-17.

Rademacher, O., Göbel, H., Ruck, M. & Oppermann, H. (2001). *Z. Krist. - New Cryst. St.* **216**, 29-30.

Radosavljevic Evans, I., Tao, S., Irvine, J. T. S. & Howard, J. A. K. (2002). *Chem. Mater.* **14**, 3700-3704.

Redrup, K. V. & Weller, M. T. (2009). *Dalton Trans.*, 4468-4472.

Sasaki, T., Ukyo, Y., Kuroda, K., Arai, S., Muto, S. & Saka, H. (2004). *J. Ceram. Soc. Jpn.* **112**, 440-444.

Weber, M., Schlesinger, M., Walther, M., Zahn, D., Schalley, C. A. & Mehring, M. (2017). *Z. Kristallogr. Cryst. Mater.* **232**, 185-207.

Yang, X., Juhas, P., Farrow, C. L. & Billinge, S. J. (2014). *arXiv preprint arXiv:1402.3163*.
